# A Time-Series Analysis of Firearm Purchasing After Mass Shooting Events in the United States

**DOI:** 10.1001/jamanetworkopen.2019.1736

**Published:** 2019-04-05

**Authors:** Gina Liu, Douglas J. Wiebe

**Affiliations:** 1 Department of Social Policy and Intervention, University of Oxford, Oxford, United Kingdom; 2 Department of Biostatistics, Epidemiology, and Informatics, University of Pennsylvania, Philadelphia

## Abstract

**Importance:**

Increased understanding of public response to mass shootings could guide public health planning regarding firearms.

**Objectives:**

To test the hypothesis that mass shootings are associated with gun purchasing in the United States and to determine factors associated with gun purchasing changes.

**Design and Setting:**

In a cross-sectional study, monthly data on US background checks for all firearm purchases, handgun permits, and long gun permits between November 1, 1998, and April 30, 2016, were obtained from the National Instant Criminal Background Check System. All mass shootings resulting in 5 or more individuals injured or killed during the study period were also identified. Interrupted autoregressive integrated moving average time-series modeling was used to identify events associated with changes in gun purchase volume. Then, logistic regression was used to identify event characteristics associated with changes in gun purchases. Analyses were performed between June 6, 2016, and February 5, 2019.

**Exposures:**

For the time-series analysis, each mass shooting was modeled as an exposure. In the logistic regression, examined factors were the shooter’s race/ethnicity, the region in the United States in which a shooting occurred, whether a shooting was school related, fatalities, handgun use, long gun use, automatic or semiautomatic gun use, media coverage level, and state political affiliation.

**Main Outcomes and Measures:**

Identification of major mass shootings significantly associated with changes in gun purchases, and the identification of event-specific factors associated with changes in gun purchases.

**Results:**

Between November 1998 and April 2016, 124 major mass shootings and 233 996 385 total background checks occurred. A total of 26 shootings (21.0%) were associated with increases in gun purchases and 22 shootings (17.7%) were associated with decreases in gun purchasing. Shootings receiving extensive media coverage were associated with handgun purchase increases (odds ratio, 5.28; 95% CI, 1.30-21.41; *P* = .02). Higher-fatality shootings had an inverse association with handgun purchase decreases (odds ratio, 0.73; 95% CI, 0.53-1.00; *P* = .049).

**Conclusions and Relevance:**

The findings of this study suggest an association between mass shootings and changes in gun purchases, observed on a comprehensive timescale. Identification of media coverage and fatalities as significant factors underlying this association invites further study into the mechanisms driving gun purchase changes, holding implications for public health response to future gun violence.

## Key Points

**Question:**

Are mass shootings associated with changes in gun purchasing, and what factors determine that association?

**Findings:**

This cross-sectional study found through interrupted time-series modeling that 21% of major mass shootings between 1998 and 2016 were significantly associated with increases in gun purchases, and 18% of mass shootings during that period were associated with decreases in gun purchases. Mass shootings receiving extensive media coverage were more likely to be associated with increases in handgun purchase, and high-fatality shootings were more likely to be associated with decreases in handgun purchases.

**Meaning:**

This study’s findings suggest that, because gun ownership frequently changes in association with mass shootings, the public health community should examine the mechanisms underlying these changes.

## Teaser

This cross-sectional study using time-series analysis tests the hypothesis that mass shootings are associated with gun purchasing in the United States and examines factors associated with changes in gun purchasing.

## Introduction

Gun violence in the United States constitutes a serious public health crisis, causing more than 30 000 deaths annually.^1^ In 2017, more deaths were attributable to firearm injuries than to motor vehicle traffic crashes (12.2 vs 11.9 deaths per 100 000 people).^2^ Mass shootings contribute to only a small fraction of this mortality and morbidity burden, at less than 1% of US firearm deaths.^3^ Nevertheless, these events offer an important lens for understanding connections between gun violence and public opinion, with implications for gun violence prevention as a whole.

Although relatively rare compared with other forms of gun violence, mass shootings are extremely high profile.^4^ These shootings often receive high media coverage; for instance, mass shootings such as the Sandy Hook Elementary School shooting were voted the top news topic of 2012 by the Associated Press, higher even than the presidential election.^5^ As a result, the fear that mass shootings inspire in the public has been disproportionate to their frequency.^6,7^ Surveys have shown that most US citizens view mass shootings as indications of underlying societal issues, which have the power to alter people’s fear of victimization and perceptions of gun control policies.^8,9,10^

Changes in gun purchases are one way in which these attitude changes after mass shootings may translate to behavior. Sharp increases in gun purchases after mass shootings, including shootings in Newtown, Connecticut; San Bernardino, California; Orlando, Florida; and Parkland, Florida, have been documented by the media.^11,12,13,14^ Empirical studies of this association have been narrower in scope but offer evidence that such an association may exist. Studies specifically examining the 2012 Sandy Hook Elementary School shooting and the 2015 San Bernardino shooting have demonstrated large increases in the volume of gun sales after the events, with excess purchases of up to 3 million guns nationally after the Sandy Hook Elementary School shooting.^15,16^ Another study observing a larger sample, yet limited to 6 highly publicized mass shootings, also found evidence that such an association between mass shootings and gun purchasing was possible.^17^ Because these studies’ scopes are limited to select shootings, especially those receiving the most extensive media coverage, there is a need for empirical examination of these effects on a larger timescale and using a broader sample of shootings.

The first of 2 main hypotheses for the increase in gun sales associated with mass shootings is that people will buy additional guns because of increased fear of victimization. Most US citizens believe that it is somewhat or very likely that their own community could experience a mass shooting.^18^ Because personal protection is the most common reason for gun ownership cited by US gun owners, it is plausible that the increased anxiety from these events could also drive increased gun ownership.^19^ However, the evidence is mixed on whether perceived risk of victimization actually translates to gun purchases.^20,21^ Increases in gun sales were observed after the September 11, 2001 (9/11), World Trade Center attacks, an event that provoked fear nationally.^22^ However, while surveys of gun owners and nonowners after the 2016 Orlando Pulse nightclub shooting demonstrated that nonowners perceived increased risk, neither group reported significantly increased gun purchasing intentions.^21^

The second main mechanism by which gun purchases might increase in response to a mass shooting is that people will buy additional guns if they believe that gun control measures will restrict their future ability to do so. Mass shootings are frequently followed by calls for gun control, whether from politicians (eg, President Barack Obama’s mention of the Sandy Hook Elementary shooting in his 2013 State of the Union address) or from the public (including student-led activism after the Marjory Stoneman Douglas High School shooting).^23,24^ In response, people concerned about potential legislation resulting from this advocacy may be motivated to buy guns or related paraphernalia, especially those that might be restricted. For example, after the 2017 Las Vegas Route 91 Harvest festival shooting, gun stores and distributors also received much higher demands for bump stock accessories of the same types used by the shooter, which were eventually banned in December 2018.^25,26^ Similar increases in purchases have been empirically observed in response to the 2008 and 2012 elections of a Democratic president^27^; the reverse effect, decreases in gun purchasing after the 2016 presidential election (colloquially termed the “Trump slump”), has also been reported, with the reasoning being that gun legislation is less likely to occur with a conservative president and Congress.^28,29^

Changes in gun purchasing behavior can have serious associations with morbidity and mortality. For instance, states that experienced increases in firearm sales after the Sandy Hook Elementary School shooting also experienced significant increases in accidental firearm deaths.^16^ These associations could potentially occur across all forms of gun violence, as associations between increased availability of guns and increased risk of suicide, homicide victimization, and unintentional injury have been observed.^30,31,32^ This association is not without complication, however, as there has been mixed evidence on whether rates of gun ownership are associated with rates of crime.^33,34^ In addition, there may be moderating factors between gun purchasing and injury, such as whether the purchaser is a new or existing gun owner.^35^

We aimed to identify, by examining more than 100 major mass shootings that took place in the United States during the past 2 decades, whether these shootings were associated with significant changes in gun purchasing behavior. We also sought to identify which characteristics of the mass shootings were associated with these changes.

## Methods

### Data Sources

We obtained data from the National Instant Criminal Background Check System on the number of background checks completed between November 1, 1998, and April 30, 2016.^36^ This database is maintained by the Federal Bureau of Investigation and used by Federal Firearms Licensees, as well as private sellers in some states, to determine customer eligibility for firearm purchases.^9^ Although not all gun purchases are subject to background checks under federal law, and even successful background checks may not always lead to purchases, background check data have been used extensively in the literature as a proxy for intent to purchase firearms.^16,17,27^ Data are aggregated monthly for all US states and territories, including such categories as permit purchases, redemptions, returns, or rentals of handguns or long guns. In this analysis we considered background checks for all gun-related purchases, handgun permits, and long gun permits on a national basis. Although background checks for handgun permits and long gun permits are included within the total background checks category, we chose to also examine these 2 categories separately to disentangle the 2 hypothesized mechanisms for changes in gun purchases. Gun buyers motivated by fear of crime are especially likely to buy handguns^37,38^; conversely, because gun control efforts after mass shootings frequently single out assault-style weapons, especially semiautomatic rifles such as the AR-15, gun buyers motivated by fear of gun control might be more likely to buy long guns.^39,40,41^ This study followed the Strengthening the Reporting of Observational Studies in Epidemiology (STROBE) reporting guideline. This study was considered exempt from review by the University of Pennsylvania Institutional Review Board because the data were aggregated and anonymized.

We obtained data on mass shooting events from the Stanford Mass Shootings in America database, which contains information on all shootings with 3 or more individuals injured or killed between August 1966 and April 2016 (as of June 2016 data collection).^10^ We included only shootings occurring since November 1998, ensuring the same timeframe as the National Instant Criminal Background Check System background checks data. We also used a narrower definition of *major mass shooting*: 5 or more individuals injured or killed. This higher morbidity threshold limited the sample to a feasible number of interruptions for the autoregressive integrated moving average (ARIMA) modeling step (N = 124); it also increased sample specificity to events that would feasibly reach the public’s attention as a mass shooting (as the public’s reaction to a mass shooting might be different from their reaction to gang-related violence, for instance) but still remained sensitive enough to capture a large number of events.

Three nonshooting events were also included in the analysis: the 9/11 World Trade Center attacks, the November 2008 presidential election of Barack Obama, and President Obama’s November 2012 reelection. These events were included because of their national significance, extensive media coverage, and literature demonstrating associated changes in gun purchasing after they took place.^22,27^ Failing to control for these major events in this way could have hampered our ability to test with accuracy whether mass shootings during the same general period were associated with changes in firearm purchasing behavior.

We obtained data on media coverage for each shooting from the LexisNexis Academic Newspapers & Wires database, a widely used news archive providing coverage of major US and international newspapers.^42,43^ For each event, we searched for newspaper articles published within 1 month of the event, recording the number of articles returned. The parameters used were the title of shooting used in the database of Stanford University of California (eg, “Sandy Hook elementary school”) “AND shoot! AND gun!” omitting generic place descriptors such as “building” on a case-by-case basis to ensure result relevance. The maximum number of results returned by the LexisNexis search was 1000 articles.

### Statistical Analysis

#### Time-Series Analysis

Seasonal ARIMA modeling was used to identify events associated with concurrent changes in the volume of all 3 background check categories: total gun purchase related, handgun permit, and long gun. ARIMA modeling is a form of interrupted time-series analysis, widely used in the public health intervention literature and considered one of the strongest possible quasiexperimental designs.^44,45^ The iterative modeling process consisted of first identifying the ARIMA(p, d, q)(P, D, Q)_m_ model that best fit the background checks time-series, in which the (p, d, q) parameters refer to the order and differencing degree of nonseasonal autoregressive and moving-average components and the (P, D, Q)_m_ parameters refer to the order and differencing degree of seasonal components, as well as the period of seasonality (m).^46^

We then used indicator variables for each mass shooting (“interruption”) to test the null hypothesis that each event was not associated with a significant change relative to the forecasted change in the volume of background checks; the multi-interruption approach that we used is similar to that used in the analysis by Wallace^17^ of select mass shootings, as well as intervention evaluations in other public health areas.^47,48^ Interruption effect patterns were tested using the following 5 different transfer functions testing different temporal patterns of gun purchase changes, which were informed by previous studies and mechanistic hypotheses: (1) zero-order function to a step variable (immediate permanent effect), (2) a first-order “pulse” function with a 3-month duration (immediate temporary effect lasting 3 months), (3) a first-order pulse function with a 5-month duration (immediate temporary effect lasting 5 months), (4) a first-order pulse function with a 5-month duration and a 1-month lag (immediate temporary effect lasting 5 months, starting 1 month after event occurrence), and (5) a combination of 2 first-order pulses in opposite directions of a total 5-month duration (gradual effect and dissipation over 5 months). The best-fit transfer function was selected based on the lowest Akaike information criterion value. Interruptions were added stepwise chronologically to the ARIMA model and retained if the *P* value coefficient remained *P* < .20. Because of the monthly nature of the background check outcome data, only 1 interruption was tested per month with multiple shootings and 1 effect size obtained for all shootings in that month. Regardless of significance, events occurring the month immediately after an event with the maximum level of media coverage (≥1000 articles) were excluded unless they also had the maximum level, as a longer-duration effect was anticipated from events with the highest level of media coverage. After modeling, event interruptions with *P* ≥ .10 were removed from the final model in order to achieve greater statistical power.

#### Logistic Regression

After the time-series analysis, we used logistic regression to identify demographic, firearm-related, and event-specific characteristics associated with changes in gun purchasing. Tested outcomes were whether a given shooting was significantly associated with any change, an increase, or a decrease in total, handgun permit, or long gun permit background checks. Tested factors were the shooter’s race/ethnicity, the region of the United States in which a shooting occurred, whether a shooting was school related, the number of fatalities, use of handguns, use of long guns, use of automatic or semiautomatic guns, whether a shooting received the highest degree of media coverage (as measured by ≥1000 articles returned by a LexisNexis search), and the political party of the governor at the time in the state in which a shooting occurred (as a proxy for regional political affiliations). Missing or unknown values were coded as NA (not applicable) values and not included in the analyses. All *P* values were from 2-sided tests and results were deemed statistically significant at *P* < .05, and multivariate logistic regression was planned in the event that multiple factors were found to be significant for a given outcome. Data analyses were performed in R (R Project for Statistical Computing) between June 6, 2016, and February 5, 2019.

## Results

**Figure 1.**
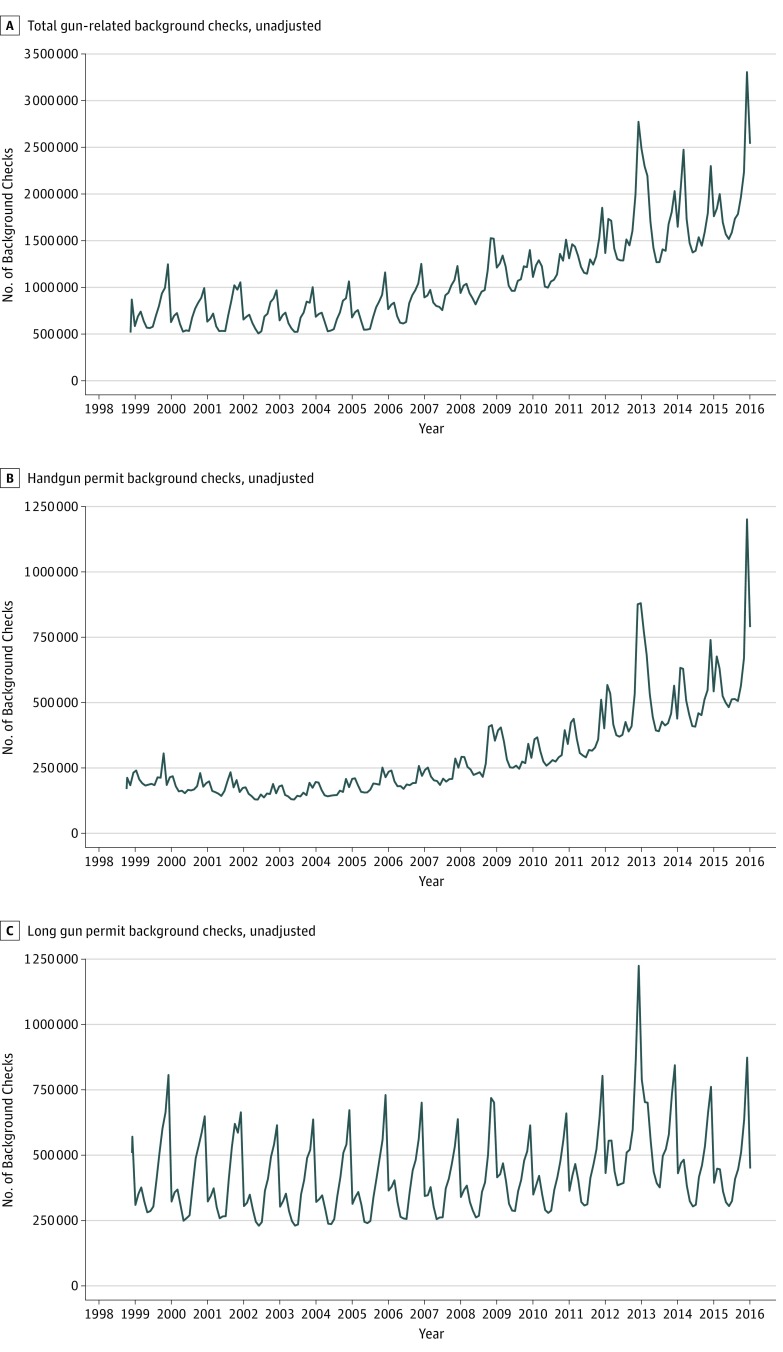
Crude Time Series for Total, Handgun Permit, and Long Gun Permit Background Checks

A total of 233 996 385 background checks occurred during the study period (Figure 1), with an upward trend exhibited in all 3 examined background check categories: total gun-related purchases, handgun permits, and long gun permits. These data also displayed strong seasonality, with annual peaks typically occurring in late fall. A total of 124 mass shootings occurred during the study period (Table 1).

**Table 1.** Summary by Selected Characteristics of the Examined Major Mass Shootings

Characteristic	No. (%) (N = 124)
Race/ethnicity of shooter	
White or European American	52 (41.9)
Black or African American	34 (27.4)
Asian American	7 (5.6)
Native American or Alaska Native	3 (2.4)
Other race/ethnicity	11 (8.9)
≥2 Races/ethnicities	2 (1.6)
Unknown	15 (12.1)
School-related shooting	
Yes	20 (16.1)
No	99 (79.8)
Unknown	5 (4.0)
Geographical region	
South	43 (34.7)
West	32 (25.8)
Midwest	31 (25.0)
Northeast	18 (14.5)
Fatalities	
≥5	67 (54.0)
1-4	39 (31.5)
None	18 (14.5)
Use of handguns	
Yes	84 (67.7)
No	16 (12.9)
Unknown	24 (19.4)
Use of long guns	
Yes	39 (47.6)
No	59 (31.5)
Unknown	26 (21.0)
Use of automatic or semiautomatic guns	
Yes	75 (60.5)
No	9 (7.3)
Unknown	40 (32.3)
Media coverage within 1 mo, No. of articles	
<500	108 (87.1)
500-999	6 (4.8)
≥1000	10 (8.1)
Political party of governor in state where shooting occurred	
Democrat	52 (58.1)
Republican	72 (41.9)

**Figure 2.**
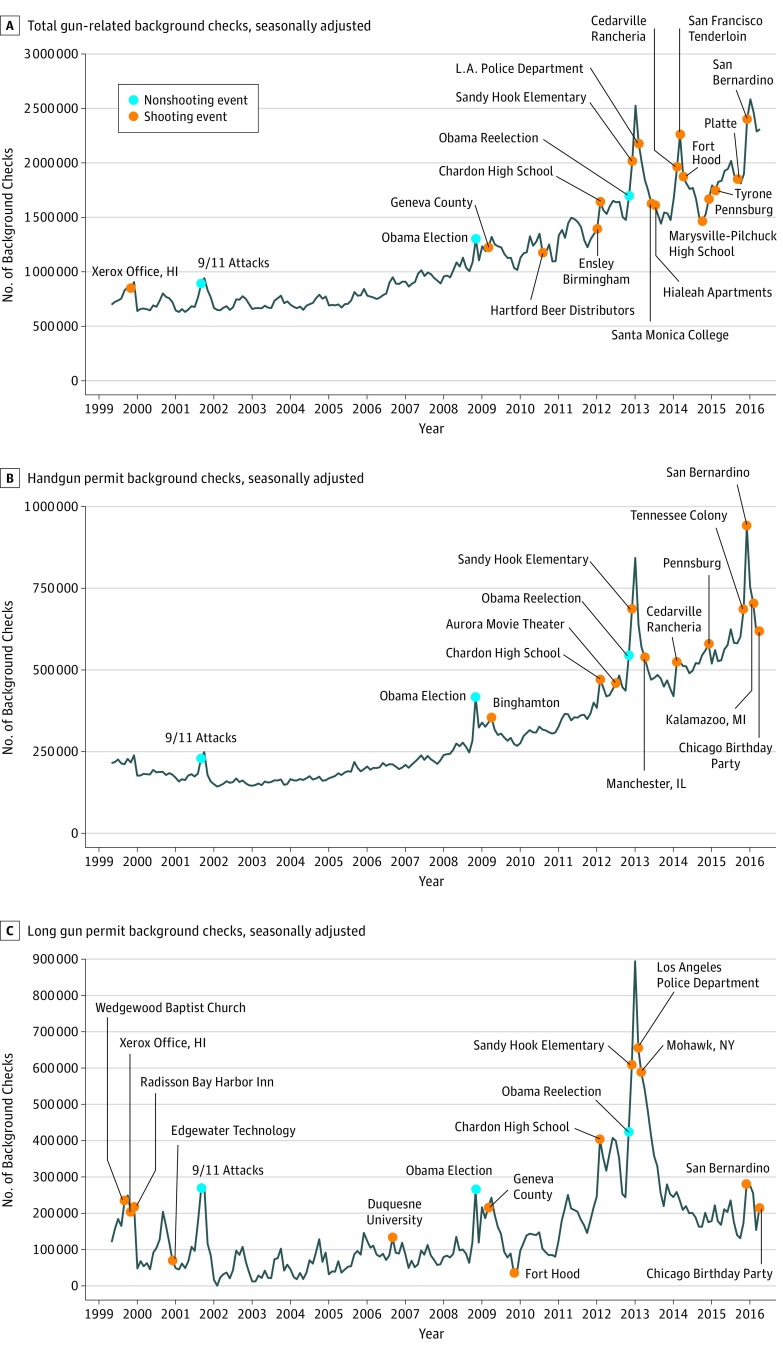
Seasonal Autoregressive Integrated Moving Average Models for Total, Handgun Permit, and Long Gun Permit Background Checks Events associated with significant gun purchase changes are labeled (n = 51).

The full form and parameters of the best-fit ARIMA models for each type of background check examined can be found in Table 2. The selected transfer function used to model interruptions was a first-order pulse function with a 5-month duration (immediate temporary effect lasting 5 months). In total, 51 of the 127 tested events (40.2%; 48 shootings [38.7%]) were associated with changes in 1 or more background check categories (Figure 2).

**Table 2.** ARIMA Models and Events Significantly Associated With Changes in Total Gun Purchase, Handgun Permit, and Long Gun Permit Background Checks

Included Event (I_i_)^a^	Month and Year	Mean (SE) Change in Gun Purchases (ω_i_)	*P* Value	Noise Model
**Total Gun Purchases (n = 28)**				
Xerox Office Building shooting, Honolulu, HI	November 1999	156 786.78 (67 241.97)	.01	ARIMA(0, 1, 0)(0, 1, 1)_12_; (1 − B)(1 − B^12^)Y_t_ = (1 − Θ_1_B^12^)a_t_; Θ_1_ = −0.0931, *P* = .20
9/11 Attacks	September 2001	92 370.06 (52 746.94)	.04	
Presidential election (2008)	November 2008	325 831.19 (53 217.05)	<.001	
Geneva County, AL, shooting; Rivermark shooting, Santa Clara, CA; and Pinelake Health and Rehabilitation Nursing Home shooting, Carthage, NC	March 2009	11 5014.5 (53 396.9)	.02	
Hartford Beer Distributors shooting, Manchester, CT	August 2010	−108 209.14 (53 079.79)	.02	
Ensley Highlands shooting, Birmingham, AL	January 2012	−275 243.09 (55 935.05)	<.001	
Chardon High School shooting, Chardon, OH	February 2012	175 028.33 (64 581.71)	.004	
President Obama reelection (2012)	November 2012	243 815.45 (54 157.81)	<.001	
Sandy Hook Elementary School shooting, Newtown, CT	December 2012	555 411.09 (53 912.86)	<.001	
Los Angeles Police Department shooting and Ladera Ranch, CA shooting	February 2013	−165 522.95 (77 891.84)	.02	
Santa Monica College shooting, Santa Monica, CA	June 2013	−123 504.57 (53 767.27)	.01	
Hialeah Apartments shooting, Hialeah, FL	July 2013	−83 221.03 (54 353.55)	.06	
Cedarville Rancheria Tribal Headquarters shooting, Cedarville, CA	February 2014	224 893.83 (77 974.13)	.002	
San Francisco Tenderloin district shooting, San Francisco, CA	March 2014	356 026.5 (54 065.0)	<.001	
Fort Hood military base shooting, Killeen, TX, and FedEx shooting, Kennesaw, GA	April 2014	−30 5053.78 (54 260.73)	<.001	
Marysville Pilchuck High School shooting, Marysville, WA	October 2014	−88 128.06 (54 276.28)	.05	
Montgomery County, PA, shooting, Pennsburg, Souderton, Lansdale, and Harleyville, PA	December 2014	245 324.93 (72 903.76)	<.001	
McAvan’s Pub shooting, Syracuse, NY; Douglasville, GA, shooting; and Tyrone, MO, shooting	February 2015	−133 182.29 (68 849.47)	.03	
Platte, SD, shooting and Hill Haven Event Center shooting, Greenville, GA	September 2015	179 574.18 (73 792.28)	.008	
San Bernardino, CA, shooting and Omaha, NE, shooting	December 2015	872 663.84 (98 856.79)	<.001	
**Handgun Purchases (n = 26)**				
9/11 Attacks	September 2001	68 064.37 (18 857.66)	<.001	ARIMA(3, 1, 0)(0, 1, 1)_12_; (1 − B)(1 − B^12^)Y_t_ = (1 − Θ_1_B^12^)/ (1 − ϕ_1_B − ϕ_2_B^2^ − ϕ_3_B^3^)a_t_; Θ_1_ = −0.3879, *P* < .001; ϕ_1_ = −0.7051, *P* < .001; ϕ_2_ = −0.2258, *P* = .03; ϕ_3_ = −0.0304, *P* = .38
Presidential election (2008)	November 2008	129 894.18 (19 249.89)	<.001	
Immigration Services Center shooting, Binghamton, NY	April 2009	367 69.45 (19 317.39)	.03	
Chardon High School schooting, Chardon, OH	February 2012	67 258.88 (19 590.65)	<.001	
Movie theater shooting, Aurora, CO	July 2012	43 555.85 (20 483.82)	.02	
President Obama reelection (2012)	November 2012	69 004.86 (25 438.41)	.003	
Sandy Hook Elementary School shooting, Newtown, CT	December 2012	361 436.25 (26 767.06)	<.001	
Village of Manchester, IL, shooting	April 2013	32 760.11 (20 671.02)	.06	
Cedarville Rancheria Tribal Headquarters shooting, Cedarville, CA	February 2014	100 410.94 (19 315.31)	<.001	
Montgomery County, PA, shooting, Pennsburg, Souderton, Lansdale, Harleyville, PA	December 2014	65 592.84 (22 989.70)	.002	
Tennessee Colony, TX, shooting and Minneapolis, MN, shooting	November 2015	122 474.56 (31 859.92)	<.001	
Planned Parenthood shooting, Colorado Springs, CO	November 2015			
San Bernardino, CA, shooting and Omaha, NE, shooting	December 2015	344 105.74 (34 698.68)	<.001	
Tampa, FL, shooting; Rochester nightclub shooting, Rochester, NY; Orlando, FL, shooting; Kalamazoo, MI, shooting; Houston, TX, drive-by shooting; Hesston, KS, shooting; and strip club shooting, Detroit, MI	February 2016	−60 154.11 (30 970.93)	.03	
Birthday party bus shooting, Chicago, IL; music video filming shooting, Chicago, IL; Halifax County, VA, shooting; and Forestville, MD, shooting	April 2016	60 243.83 (35 802.34)	.05	
**Long Gun Purchases (n = 23)**				
Wedgwood Baptist Church shooting, Fort Worth, TX	September 1999	71 717.53 (34 028.21)	.02	ARIMA(0, 1, 0)(1, 1, 1)_12_; (1 − B)(1 − B^12^)Y_t_ = (1 − Θ_1_B^12^)/(1 − Φ_1_B^12^)a_t_; Θ_1_ = 0.5765, *P* < .001; Φ_1_ = −0.7613, *P* < .001
Xerox Office Building shooting, Honolulu, HI	November 1999	111 571.45 (32 285.38)	<.001	
Radisson Bay Harbor Inn shooting, Tampa, FL	December 1999	−151 579.31 (29 658.04)	<.001	
Edgewater Technology shooting, Wakefield, MA	December 2000	−157 279.09 (27 724.69)	<.001	
9/11 Attacks	September 2001	66 032.65 (26 485.92)	.006	
Duquesne University shooting, Pittsburgh, PA	September 2006	33 892.88 (25 397.95)	.09	
Presidential election (2008)	November 2008	145 444.96 (27 463.29)	<.001	
Geneva County, AL, shooting; Rivermark shooting, Santa Clara, CA; and Pinelake Health and Rehabilitation Nursing Home shooting, Carthage, NC	March 2009	41 272.98 (25 720.82)	.05	
Fort Hood Army Base shooting, Killeen, TX	November 2009	−42 864.97 (27 526.48)	.06	
Chardon High School shooting, Chardon, OH	February 2012	69 056.38 (27 276.71)	.006	
President Obama reelection (2012)	November 2012	132 521.49 (26 574.07)	<.001	
Sandy Hook Elementary School shooting, Newtown, CT	December 2012	263 346.05 (26 700.57)	<.001	
Los Angeles Police Department shooting and Ladera Ranch, CA, shooting	February 2013	−48 703.25 (27 543.76)	.04	
Mohawk Village and Herkimer Village, NY, shooting	March 2013	54 162.67 (25 985.47)	.02	
San Bernardino, CA, shooting and Omaha, NE, shooting	December 2015	135 638.1 (35 809.0)	<.001	
Birthday party bus shooting, Chicago, IL; music video filming shooting, Chicago, IL; Halifax County, VA, shooting; and Forestville, MD, shooting	April 2016	77 161.4 (38 076.09)	.02	

Abbreviations: ARIMA, autoregressive integrated moving average; a_t_, random error; B, backward shift operator; I_i_, given interruption; Y_t_, current time-series observation; Θ, seasonal moving-average operator; ϕ, autoregressive operator; Φ, seasonal autoregressive operator; ω_i_, ARIMA coefficient corresponding to I_i_.

^a^ When more than 1 event occurred in the same month, only 1 effect size was calculated per month. The intervention model for all included events is Y_t_* = a_t_ + Σ(I_i_ × ω_i_).

A total of 29 events (22.8%; 26 shootings [21.0%]) were associated with increases in 1 or more categories of background checks. Seven events were associated with increases in all categories; these included the 3 nonshooting events (9/11 attacks and the 2008 and 2012 presidential elections), Sandy Hook Elementary School shooting, and San Bernardino shooting. Two events were associated with increases in total gun-related checks, 4 were associated with increases in total and long gun permit checks, and 4 were associated with increases in handgun and long gun permit checks. Three events were associated with increases in only total gun-related checks, 6 were associated with increases in only handgun permit checks, and 3 were associated with increases in only long gun permit checks.

A total of 22 events (17.3% of all 127 events; 17.7% of all 124 shootings) were associated with decreases in 1 or more categories of background checks. Two events were associated with decreases in total and long gun permit checks. Ten events were associated with decreases in only total checks, 7 were associated with decreases in only handgun permit checks, and 3 were associated with decreases in only long gun permit checks.

None of the examined characteristics was found to be significantly associated with changes, whether negative or positive, in purchases of long guns. High media coverage, defined as 1000 or more articles published about a shooting within 1 month of the shooting, was significantly associated with an increase in handgun permits (odds ratio, 5.28; 95% CI, 1.30-21.41; *P* = .02). The number of fatalities from a shooting was significantly associated with a decrease in handgun permits, with an increase of 1 fatality from a shooting reducing the odds of handgun permit decreases by a factor of 0.73 (95% CI, 0.53-1.00; *P* = .049). Logistic regression model results are included in Table 3.

**Table 3.** Unadjusted Logistic Regression Odds Ratios to Identify Factors Associated With Shootings Associated With Any Significant Change, an Increase, or a Decrease in Gun Purchases

Factor	Odds Ratio (95% CI)								
	Total Background Checks			Handgun Permit Background Checks			Long Gun Permit Background Checks		
	All Changes	Increase	Decrease	All Changes	Increase	Decrease	All Changes	Increase	Decrease
Race/ethnicity of shooter									
White or European American	1 [Reference]	1 [Reference]	1 [Reference]	1 [Reference]	1 [Reference]	1 [Reference]	1 [Reference]	1 [Reference]	1 [Reference]
Black or African American	1.29 (0.45-3.69)	0.81 (0.19-3.51)	2.02 (0.50-8.21)	0.64 (0.18-2.26)	0.36 (0.07-1.81)	2.87 (0.25-33.07)	0.73 (0.20-2.66)	0.73 (0.17-3.16)	0.73 (0.06-8.45)
Other or unknown	0.95 (0.32-2.77)	0.90 (0.23-3.48)	1.02 (0.21-4.87)	1.71 (0.62-4.73)	1.15 (0.36-3.68)	6.14 (0.65-57.84)	1.47 (0.50-4.34)	1.47 (0.43-4.98)	1.47 (0.20-10.99)
Region of shooting									
Northeast	1 [Reference]	1 [Reference]	1 [Reference]	1 [Reference]	1 [Reference]	1 [Reference]	1 [Reference]	1 [Reference]	1 [Reference]
Midwest	0.52 (0.11-2.39)	0.78 (0.12-5.21)	0.26 (0.02-3.11)	1.43 (0.37-5.55)	1.27 (0.27-5.93)	1.91 (0.18-20.22)	0.52 (0.11-2.39)	0.69 (0.14-3.53)	Omitted^a^
South	0.80 (0.21-3.09)	0.60 (0.09-3.99)	1.00 (0.17-5.77)	0.57 (0.14-2.32)	0.38 (0.07-2.10)	1.14 (0.11-11.85)	0.68 (0.17-2.69)	0.65 (0.14-3.08)	0.78 (0.07-9.27)
West	1.37 (0.35-5.29)	1.52 (0.26-8.93)	1.22 (0.20-7.53)	0.50 (0.11-2.30)	0.67 (0.13-3.40)	Omitted^a^	0.65 (0.15-2.80)	0.52 (0.09-2.91))	1.04 (0.09-12.45)
School-related shooting	0.62 (0.17-2.30))	0.82 (0.17-4.06)	0.41 (0.05-3.41)	0.92 (0.24-3.49)	1.46 (0.36-5.89)	Omitted^a^	1.07 (0.28-4.14)	1.67 (0.41-6.80)	Omitted^a^
No. of fatalities	1.05 (0.97-1.14)	1.08 (0.99-1.18)^b^	1.00 (0.87-1.15)	1.00 (0.91-1.10)	1.05 (0.96-1.15)	0.73 (0.53-1.00)^c^	1.06 (0.97-1.15)	1.05 (0.96-1.15)	1.06 (0.92-1.23)
Use of long guns	0.96 (0.37-2.51)	1.25 (0.35-4.47)	0.75 (0.21-2.71)	1.71 (0.48-5.40)	2.01 (0.50-8.02)	0.80 (0.07-9.21)	1.92 (0.63-5.80)	1.68 (0.45-6.26)	2.52 (0.40-15.90)
Use of handguns	1.44 (0.37-5.57)	2.27 (0.27-19.14)	1.03 (0.20-5.27)	0.95 (0.19-4.79)	0.66 (0.12-3.52)	Omitted^a^	1.28 (0.26-6.32)	1.77 (0.21-15.14)	0.79 (0.08-7.60)
Use of automatic or semiautomatic guns	1.11 (0.21-5.80)	Omitted^a^	0.49 (0.09-2.79)	0.96 (0.11-8.66)	0.84 (0.09-7.69)	Omitted^a^	0.73 (0.14-3.94)	1.02 (0.11-9.25)	0.45 (0.04-4.63)
High media coverage (≥1000 articles in 1 mo)	0.99 (0.20-4.98)	2.07 (0.39-11.00)	Omitted^a^	3.33 (0.86-12.96)	5.28 (1.30-21.41)^c^	Omitted^a^	2.45 (0.58-10.40)	2.13 (0.40-11.38)	3.46 (0.34-35.31)
State governor political party									
Republican	1 [Reference]	1 [Reference]	1 [Reference]	1 [Reference]	1 [Reference]	1 [Reference]	1 [Reference]	1 [Reference]	1 [Reference]
Democrat	1.36 (0.56-3.29)	1.26 (0.40-4.04)	1.48 (0.44-4.90)	1.08 (0.43-2.70)	1.81 (0.62-5.23)	0.23 (0.03-2.02)	1.16 (0.44-3.04)	1.24 (0.42-3.68)	0.95 (0.15-5.90)

^a^ Because of insufficient sample size. ^b^*P* < .10. ^c^*P* < .05.

## Discussion

A total of 48 major mass shootings (38.7%) in the United States from November 1998 to April 2016 were associated with changes in gun purchasing; 26 shootings were associated with increases and 22 shootings were associated with decreases. That such effects may occur has been posted extensively in media venues but has not been evaluated as a research question with the comprehensive approach performed herein, to our knowledge. The increases observed were consistent with previous studies examining smaller sets of shootings and specific political events.^15,16,17,27^ However, the decreases in gun purchases observed were unexpected based on previous literature. We also identified that the shootings that receive a high amount of media coverage are more likely to be associated with significant increases in handgun purchasing and shootings with more fatalities are more likely to be associated with significant decreases in handgun purchasing.

Background checks associated with handgun vs long gun permits were examined separately to differentiate between the 2 major hypotheses underlying increases in gun purchases. Because of the connection between handgun ownership and self-defense, the logistic regression findings are relevant primarily to the fear of victimization hypothesis.^37^ The association between media coverage and increased handgun purchases is supported by the finding by Wallace^17^ that the increase in gun purchases associated with a shooting varies with the degree of news coverage. As the media coverage that mass shootings receive is disproportionate and frequently sensationalized, it thus inspires fear and motivates gun purchases for self-defense.^49^ Our finding that shootings with more fatalities were associated with decreases in handgun purchases also lends credence to the association between media, public perception of victimization, and gun purchases; because shootings with higher fatalities tend to receive both more and longer news articles, the increased effect on the public’s anxiety might counteract a decrease in purchases of guns for self-defense that would otherwise occur.^8,50^ As the shooter’s race/ethnicity and ideological motivation are also associated with an increase in the extent of media coverage as well as with its content, media focus on the perpetrator rather than victims may also contribute to increasing handgun purchases.^51^

The association of the 22 mass shootings with decreases in 1 or more categories of gun purchases was an unexpected finding of the study. One potential hypothesis is regression to the mean, especially given that some shooting events associated with decreases in gun purchasing occurred closely after extremely high-profile shootings associated with large increases in all forms of gun purchases (eg, the Sandy Hook Elementary School shooting, which was followed 2 months later by the Los Angeles Police Department shooting by Christopher Dorner and by the Ladera Ranch, California, shooting). Because previous research finding only increases in gun purchases has focused on select mass shootings rather than a broader sample of mass shootings, it is also possible that lower-profile mass shootings may result in different changes in public perceptions, although this possibility is as yet unexplored. However, there were no shootings associated with decreases in all categories of background checks, as opposed to 7 events associated with increases in all categories, so the mechanisms underlying decreases of gun purchases may operate on a smaller scale than the mechanisms underlying increases of gun purchases.

### Limitations

One limitation of this study is that it is not possible to draw causal conclusions from the findings without more in-depth investigation and elimination of confounders. Three nonshooting events (the 9/11 World Trade Center attacks, the 2008 presidential election, and the 2012 reelection) were added as their effects on national gun purchases have been well documented.^22,27^ However, there may have been other confounding factors, which could be especially salient in explaining the observed decreases in gun purchases. For example, deregulation of firearms by state legislatures and the lack of federal firearms laws from 2008 to 2016, despite the Democratic president at the time, may have allayed fears about gun control, leading to decreases in gun purchases^52^; such associations have been observed recently since the 2016 presidential election.^29^

Because of the frequent focus on assault-style weapons in the media and gun control advocacy, it was hypothesized that use of automatic or semiautomatic guns during a shooting would increase the likelihood of increases in both handgun and long gun purchases.^53^ However, our analysis did not find a significant association between the type of gun used in a shooting and changes in any category of gun purchase. This finding may have been due to power issues from the sample of mass shootings (N = 124); as this analysis is intended as primarily hypothesis generating, we will be highlighting those estimates with larger odds ratios but nonsignificant results for future follow-up.

Because the examination of gun purchases was performed at a national level, there may have been event effects specific to region or urbanization level that were not captured. For example, the effects of a mass shooting might be greater in areas closer to the shooting location. If gun purchase behavior is most affected only among persons living locally to a given mass shooting event, that would likely have created bias in our results toward the null; associations found to be positive at the national level would likely have a larger signal had they been measured at the local level.

Although this study examined only gun purchases that could be captured by available background check data, also examining private gun sales could allow for a greater understanding of the effects of mass shootings on overall levels of gun ownership. An estimated 50% of private gun acquisitions, which occur outside of a gun shop or pawn shop, are not preceded by a background check, because fewer than half of US states extend background check requirements past the federal mandate (with varying levels of coverage).^54,55^ Google Trends could give a proxy for people’s interest in privately selling their guns, given a lack of direct measures of these data, and has previously been used as a proxy for gun acquisition.^16^

## Conclusions

Although data limitations prevented this analysis from examining the potential effects of mass shootings that occurred after 2016 (including the October 2018 shooting at the Pittsburgh Tree of Life synagogue), the 2016 election and conservative presidency, and the March for Our Lives gun control activism that began after the Marjory Stoneman Douglas High School shooting, we believe this study’s results suggest that gun purchasing behavior during later time periods may also have fluctuated based on certain key events. The analysis not only identified that a large proportion (38.7%) of the tested major mass shootings were associated with changes in gun purchase, presenting a more comprehensive look at the phenomenon, but also identified significant associations between media coverage, incident severity, and gun purchasing behavior. Given these conclusions, understanding the mechanisms underlying these changes is an important component of the public health community’s response to these shootings.
